# Identification of common genes and pathways between type 2 diabetes and COVID-19

**DOI:** 10.3389/fgene.2024.1249501

**Published:** 2024-04-18

**Authors:** Ya Wang, Kai Li, Shuangyang Mo, Peishan Yao, Jiaxing Zeng, Shunyu Lu, Shanyu Qin

**Affiliations:** ^1^ Gastroenterology Department, The First Affiliated Hospital of Guangxi Medical University, Nanning, China; ^2^ Endocrinology Department, Liuzhou Peoples’ Hospital Affiliated to Guangxi Medical University, Liuzhou, China; ^3^ Orthopedics Department, The Fourth Affiliated Hospital of Guangxi Medical University, Liuzhou, China; ^4^ Gastroenterology Department, Liuzhou Peoples’ Hospital Affiliated to Guangxi Medical University, Liuzhou, China; ^5^ Department of Traumatic Surgery, Microsurgery, and Hand Surgery, Guangxi Zhuang Autonomous Region People’s Hospital, Nanning, Guangxi, China; ^6^ Department of Pharmacy, Affiliated Tumor Hospital of Guangxi Medical University, Nanning, Guangxi, China

**Keywords:** T2DM, COVID-19, common feature genes, bioinformatics, pathways

## Abstract

**Background:**

Numerous studies have reported a high incidence and risk of severe illness due to coronavirus disease 2019 (COVID-19) in patients with type 2 diabetes (T2DM). COVID-19 patients may experience elevated or decreased blood sugar levels and may even develop diabetes. However, the molecular mechanisms linking these two diseases remain unclear. This study aimed to identify the common genes and pathways between T2DM and COVID-19.

**Methods:**

Two public datasets from the Gene Expression Omnibus (GEO) database (GSE95849 and GSE164805) were analyzed to identify differentially expressed genes (DEGs) in blood between people with and without T2DM and COVID-19. Gene ontology (GO) and Kyoto Encyclopedia of Genes and Genomes (KEGG) enrichment analyses were performed on the common DEGs. A protein-protein interaction (PPI) network was constructed to identify common genes, and their diagnostic performance was evaluated by receiver operating characteristic (ROC) curve analysis. Validation was performed on the GSE213313 and GSE15932 datasets. A gene co-expression network was constructed using the GeneMANIA database to explore interactions among core DEGs and their co-expressed genes. Finally, a microRNA (miRNA)-transcription factor (TF)-messenger RNA (mRNA) regulatory network was constructed based on the common feature genes.

**Results:**

In the GSE95849 and GSE164805 datasets, 81 upregulated genes and 140 downregulated genes were identified. GO and KEGG enrichment analyses revealed that these DEGs were closely related to the negative regulation of phosphate metabolic processes, the positive regulation of mitotic nuclear division, T-cell co-stimulation, and lymphocyte co-stimulation. Four upregulated common genes (*DHX15*, *USP14*, *COPS3*, *TYK2*) and one downregulated common feature gene (*RIOK2*) were identified and showed good diagnostic accuracy for T2DM and COVID-19. The AUC values of *DHX15*, *USP14*, *COPS3*, *TYK2*, and *RIOK2* in T2DM diagnosis were 0.931, 0.917, 0.986, 0.903, and 0.917, respectively. In COVID-19 diagnosis, the AUC values were 0.960, 0.860, 1.0, 0.9, and 0.90, respectively. Validation in the GSE213313 and GSE15932 datasets confirmed these results. The miRNA-TF-mRNA regulatory network showed that TYH2 was targeted by PITX1, PITX2, CRX, NFYA, SREBF1, RELB, NR1L2, and CEBP, whereas miR-124-3p regulates THK2, RIOK2, and USP14.

**Conclusion:**

We identified five common feature genes (*DHX15*, *USP14*, *COPS3*, *TYK2*, and *RIOK2*) and their co-regulatory pathways between T2DM and COVID-19, which may provide new insights for further molecular mechanism studies.

## 1 Introduction

Type 2 diabetes mellitus (T2DM) is a highly prevalent disease worldwide. The number of people with diabetes was 285 million globally in 2010 and 463 million in 2019, and it is estimated to reach 710 million by 2045 (Shaw et al., 2010; Grewal et al., 2020; Sharma et al., 2022). Coronavirus disease 2019 (COVID-19) is an acute respiratory disease caused by SARS-CoV-2 infection. COVID-19 is highly infectious, shows rapid mutation, and has become a global pandemic, resulting in high morbidity and mortality due to complications (To et al., 2021). Previous studies have shown a significant genetic correlation between T2DM and COVID-19 (Wu K. C. H. et al., 2022; Ni et al., 2023). Furthermore, Mendelian randomization has demonstrated a causal relationship between genetic susceptibility to T2DM and SARS-CoV-2 infection and hospitalization due to COVID-19 (Host Genetics Initiative, 2022). Studies have reported that the incidence and severity of COVID-19 are higher in patients with diabetes (Leon-Abarca et al., 2021; Sharma et al., 2022). Oxidative stress and chronic inflammation are pathological mechanisms underlying the occurrence and development of T2DM and its related chronic complications (Wronka et al., 2022). Chronic inflammation leads to insulin resistance, causing the progression of T2DM, which has pro-inflammatory characteristics. Research has shown that hyperglycemia can stimulate immune cells and increase pro-inflammatory cytokines such as tumor necrosis factor α, interleukin 1β, and interleukin −6 (IL-6) (Muniyappa and Gubbi, 2020). Inflammatory markers such as IL-6, C-reactive protein, and ferritin in the blood of diabetic patients are higher than those in non-diabetic patients (Guo et al., 2020). Inflammation and immunity are involved in the progression of diabetic nephropathy (Chen et al., 2022; Rayego-Mateos et al., 2023). Mitochondrial metabolism and immune inflammation are critical factors in the pathogenesis of diabetic cardiomyopathy (Chen et al., 2022). A retrospective cohort study showed that people with COVID-19 combined with diabetes had a more robust inflammatory immune response and increased in-hospital mortality rate due to cardiac damage (Bo et al., 2022). This indicates that inflammation and immunity are closely related to T2DM and its complications. Patients with diabetes combined with COVID-19 may be more susceptible to the cytokine storm, leading to disease exacerbation. Additionally, diabetic patients have higher levels of D-dimer compared to non-diabetic controls, indicating that they are in a hypercoagulable state (Guo et al., 2020). After COVID-19 infection, the coagulation cascade is activated, exacerbating the hypercoagulable state and leading to adverse outcomes of COVID-19.

Research has shown that COVID-19 may affect the pathophysiology of diabetes. Patients with diabetes and COVID-19 may experience elevated blood glucose, possibly due to stress (Wang W. et al., 2020). Additionally, nearly 10% of COVID-19 patients with T2DM experience hypoglycemia (Zhou et al., 2020), and COVID-19 may induce the development of diabetes in people without a history of the disease (Khunti et al., 2021). The progression and prognosis of both diabetes and COVID-19 are interrelated. However, little is known about the molecular pathways underlying the abnormal inflammatory and immune responses in patients with diabetes and COVID-19. Given the vast population of patients with T2DM combined with COVID-19, the complex relationship between T2DM and COVID-19, the lack of dual-purpose treatment strategies for these diseases, and the limited research on common pathways between T2DM and COVID-19, we employed various bioinformatics analysis methods to study the shared genes and pathways between T2DM and COVID-19, providing clues for the development of dual-purpose preventive and therapeutic strategies.

## 2 Materials and methods

### 2.1 Microarray chip data

**FIGURE 1:**
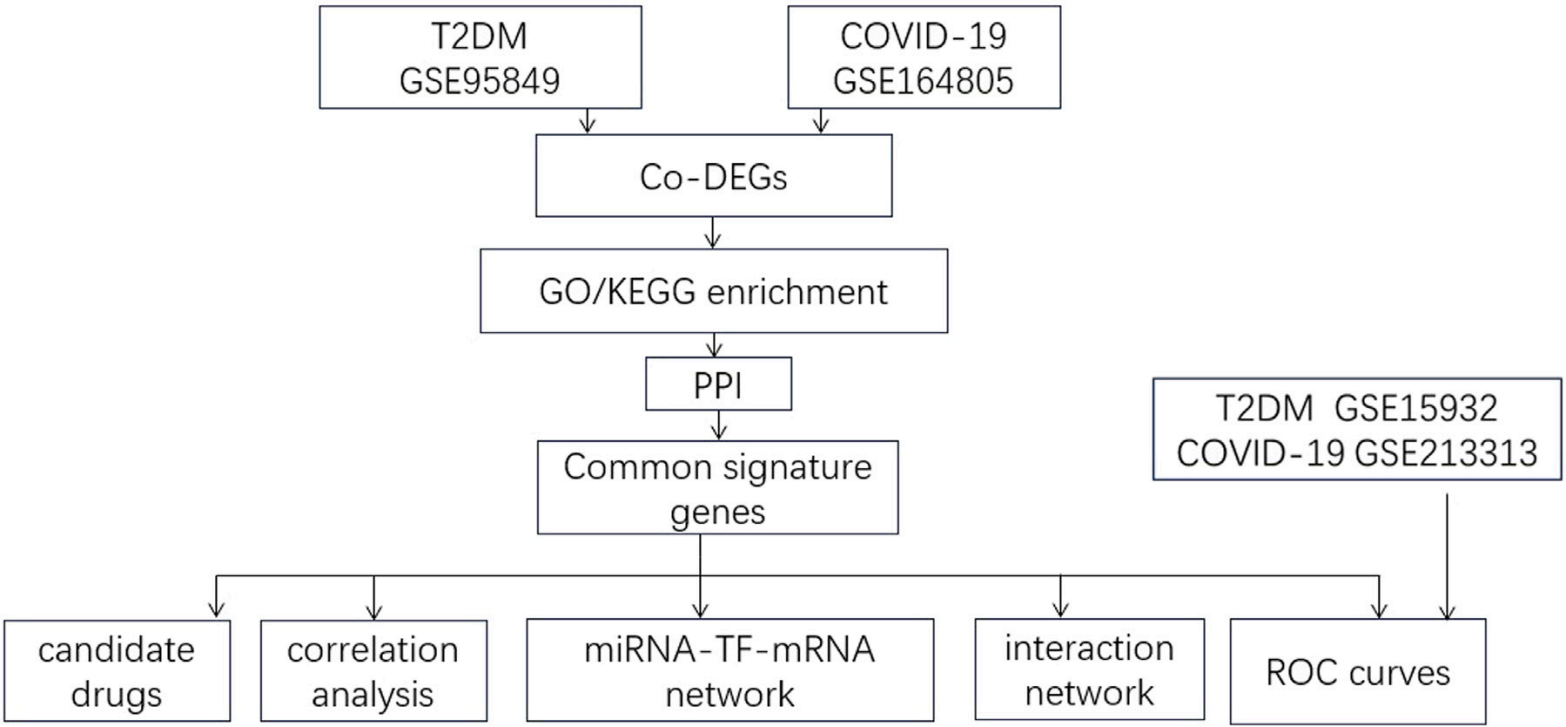
Flow chart of the study.

We downloaded datasets from the Gene Expression Omnibus (GEO) database (http://www.ncbi.nlm.nih.gov/geo) that met the following inclusion criteria: 1. Studies published between 2012 and 2023; 2. Studies on blood samples from patients with T2DM and healthy controls and those with blood samples from COVID-19 patients and healthy controls; 3. Datasets with a sample size ≥5. We excluded studies with duplicate samples and incomplete data. Two T2DM datasets were included, GSE95849 and GSE15932; two COVID-19 datasets were included, GSE164805 and GSE21331. A flowchart of this study is presented in Figure 1. GSE95849 contains mRNA expression profiles of 18 blood samples, including 12 patients with T2DM and six healthy controls. Detailed information on this dataset is shown in Table 1. GSE15932 contains mRNA expression profiles on 15 peripheral blood mononuclear cell samples, including 10 COVID-19 patients and five healthy controls. GSE213313 contains mRNA expression profiles on 94 blood samples, including 83 COVID-19 patients and 11 healthy controls. GSE15932 contains mRNA expression profiles on 16 blood samples, including eight T2DM patients and 11 healthy controls. We analyzed the GSE95849 and GSE164805 datasets and validated the results in the GSE15932 and GSE213313 datasets.

**TABLE 1:** Details of the GEO datasets.

Dataset	Disease	Platform	Organism	Number of samples
GSE95849	T2DM	GPL22448	Homo	18
GSE15932	T2DM	GPL570	Homo	16
GSE164805	COVID-19	GPL26963	Homo	15
GSE213313	COVID-19	GPL21185	Homo	94

### 2.2 Identification of differentially expressed genes shared by T2DM and COVID-19

We performed standardization and preprocessing on the data using the “limma” package in R (v.4.3.0), removed gene probes with missing values and duplicates, took the log2 transformation of the untransformed data, and conducted differential expression analysis to determine the differentially expressed genes (DEGs) between the disease groups and healthy control groups. We used the Benjamini–Hochberg method to correct the *p*-values for DEGs. We identified DEGs based on the corrected *p* < 0.05 and |log fold change (FC) |≥ 0.085. We took the intersection of the DEGs from both analyses to obtain the shared DEGs. We visualized the results using a Venn diagram.

### 2.3 Pathway enrichment analysis

Using the “clusterProfiler” package in R, we performed Gene Ontology (GO) enrichment analysis on the shared DEGs to determine enriched biological processes (BP), molecular functions (MF), and cellular components (CC). We also analyzed Kyoto Encyclopedia of Genes and Genomes (KEGG) pathways to identify shared pathways between T2DM and COVID-19. We set the filtering criteria as *p* < 0.05 and visualized the results using the “ggpubr” package.

### 2.4 Protein-protein interaction network construction and identification of common feature genes

We used the online database STRING (http://www.string-db.org/) to construct a protein-protein interaction network (PPI) to predict the functional interactions between proteins. We visualized the results using Cytoscape software and calculated Maximal Clique Centrality scores (Wu J. et al., 2022). We identified hub genes using the CytoHubba plugin in Cytoscape and assessed the most critical modules in the PPI network using the MCODE plugin in Cytoscape.

### 2.5 ROC curve analysis of common genes

To evaluate the sensitivity and specificity of the shared DEGs in diagnosing T2DM and COVID-19, we used the pROC package in R to perform receiver operating characteristic (ROC) curve analysis. We used the area under the curve (AUC) to evaluate the diagnostic performance of the common genes for T2DM and COVID-19. We also validated the diagnostic performance using ROC curve analysis in the validation dataset.

### 2.6 Interaction network of common genes

We used the GeneMANIA database (http://genemania.org/) to construct interaction networks of the five common feature genes and their co-expressed genes.

### 2.7 Correlation analysis of shared DEGs

We used the “corrplot” package in R to perform a correlation analysis of the common feature genes and calculate the correlation coefficients between genes to observe the regulatory relationships among the five common feature genes.

### 2.8 Regulatory network analysis

We used the miRTarBase (Chou et al., 2018), Starbase (Yang et al., 2011), and Targetscan (Agarwal et al., 2015) databases to predict the target microRNAs (miRNAs) of the shared core genes. We only retained miRNAs predicted by all three databases. We predicted the target transcription factors (TFs) of the shared core genes using the Enrichr website (https://maayanlab.cloud/Enrichr/) by inputting the names of the shared core genes and selecting the “TRANSFAC and JASPAR PWMS” option. We downloaded the data table, retained the TFs with a *p* < 0.05, and restricted them to genes from humans. Using Cytoscape, we visualized the miRNA-TF-messenger RNA (mRNA) regulatory network.

### 2.9 Candidate drugs

We used the DGIdb website (DGIdb—Mining the Druggable Genome) to identify candidate drugs based on the integrated score obtained by inputting the common feature genes.

## 3 Results

### 3.1 Shared DEGs between T2DM and COVID-19

**FIGURE 2:**
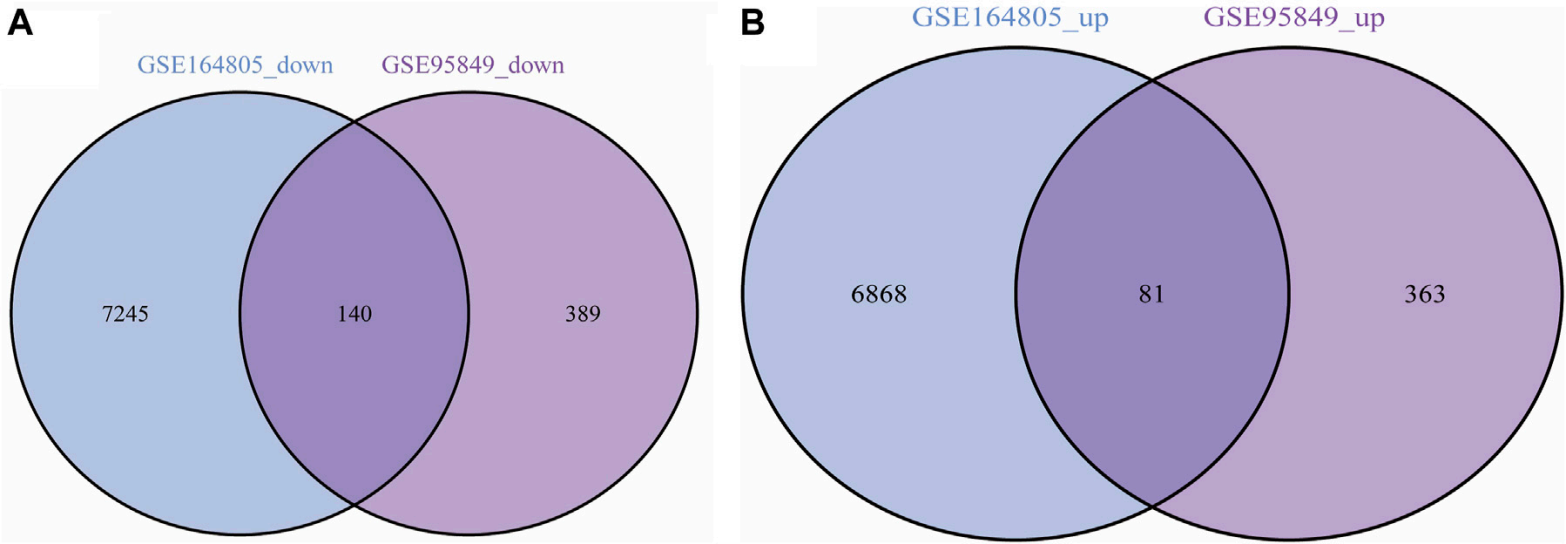
**(A)** Common upregulated genes in COVID-19 and T2DM; **(B)** Common downregulated genes in COVID-19 and T2DM.

We performed differential expression analysis and identified 973 DEGs in the T2DM dataset GSE9584 and 143,344 DEGs in the COVID-19 dataset GSE164805. We took the intersection of the DEGs from both datasets and identified 221 shared DEGs (Figure 2), including 81 upregulated genes (Figure 2B) and 140 downregulated genes (Figure 2A).

### 3.2 Functional and enrichment analysis of shared DEGs

**FIGURE 3:**
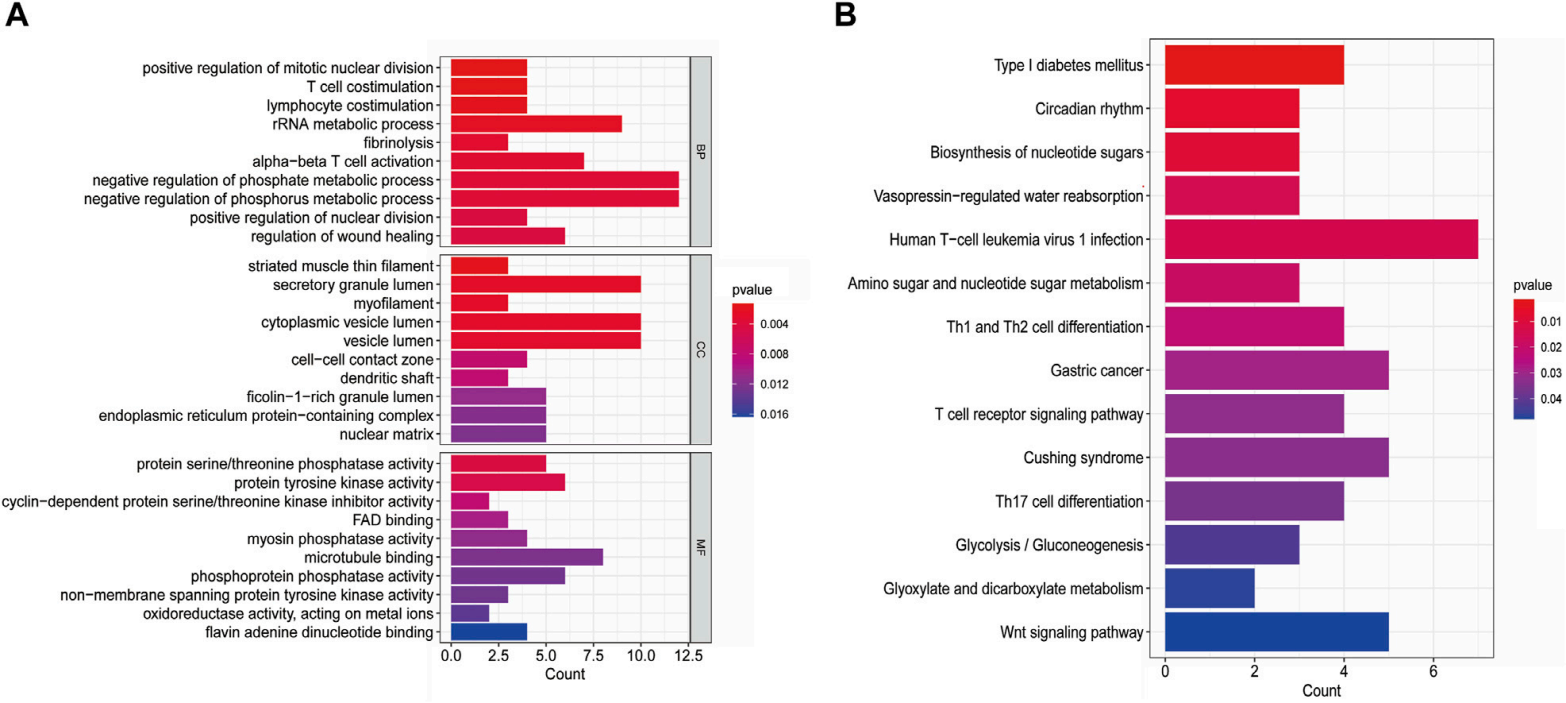
**(A)** GO pathways of shared DEGs between T2DM and COVID-19; **(B)** KEGG pathways of shared DEGs between T2DM and COVID-19.

The GO-BP results showed that these shared DEGs are mainly associated with biological processes such as “negative regulation of phosphate metabolic process,” “negative regulation of phosphorus metabolic process,” “rRNA metabolic process,” “positive regulation of mitotic nuclear division,” “T cell co-stimulation,” “lymphocyte co-stimulation,” “fibrinolysis,” and “alpha-beta T cell activation.” In the GO-CC category, the shared DEGs were enriched in areas such as “secretory granule lumen,” “cytoplasmic vesicle lumen,” “vesicle lumen,” “striated muscle thin filament,” and “myofilament.” In the GO-MF category, the shared DEGs were mainly involved in functions such as “protein serine/threonine phosphatase activity,” “protein tyrosine kinase activity,” and “microtubule binding” (Figure 3A). KEGG enrichment analysis revealed that these shared DEGs were primarily enriched in pathways such as “human T-cell leukemia virus 1 infection,” “type I diabetes mellitus,” “circadian rhythm,” “biosynthesis of nucleotide sugars,” “amino sugar and nucleotide sugar metabolism,” “Th1 and Th2 cell differentiation,” “gastric cancer,” “Cushing’s syndrome,” and “T-cell receptor signaling pathway” (Figure 3B). These results suggest that metabolism and the inflammatory response play essential roles in the crosstalk between T2DM and COVID-19.

### 3.3 PPI network and extraction of core genes

**FIGURE 4:**
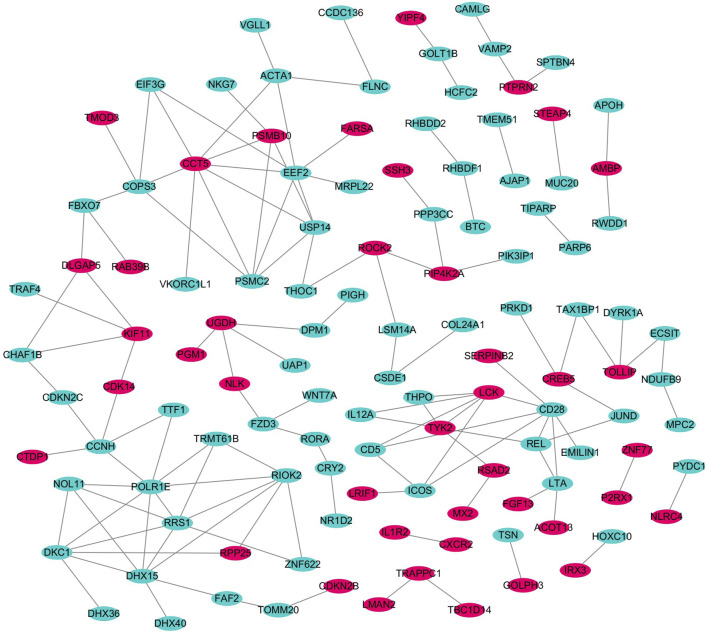
PPI network of shared DEGs.

We imported the shared DEGs of T2DM and COVID-19 into the STRING database, with a minimum required interaction source set to 0.4 and free nodes hidden, resulting in a PPI network with 219 nodes and 133 edges. We visualized the network using Cytoscape software, with red and blue nodes representing upregulated and downregulated genes, respectively (Figure 4). Using the MCODE plugin in Cytoscape, we identified five important modules with parameter settings of Degree Cutoff: 2, Node Score Cutoff: 0.2, K-Core: 2, and Max. Depth: 100. We calculated the Maximal Clique Centrality scores and subsequently used the Cytohubba plugin in Cytoscape to identify high-connectivity hub genes in the network. We identified five common feature genes between T2DM and COVID-19: *DHX15*, *USP14*, *COPS3*, *TYK2*, and *RIOK2* (where *DHX15*, *USP14*, *COPS3*, and *TYK2* were upregulated and *RIOK2* was downregulated).

### 3.4 ROC curves of common feature genes

**FIGURE 5:**
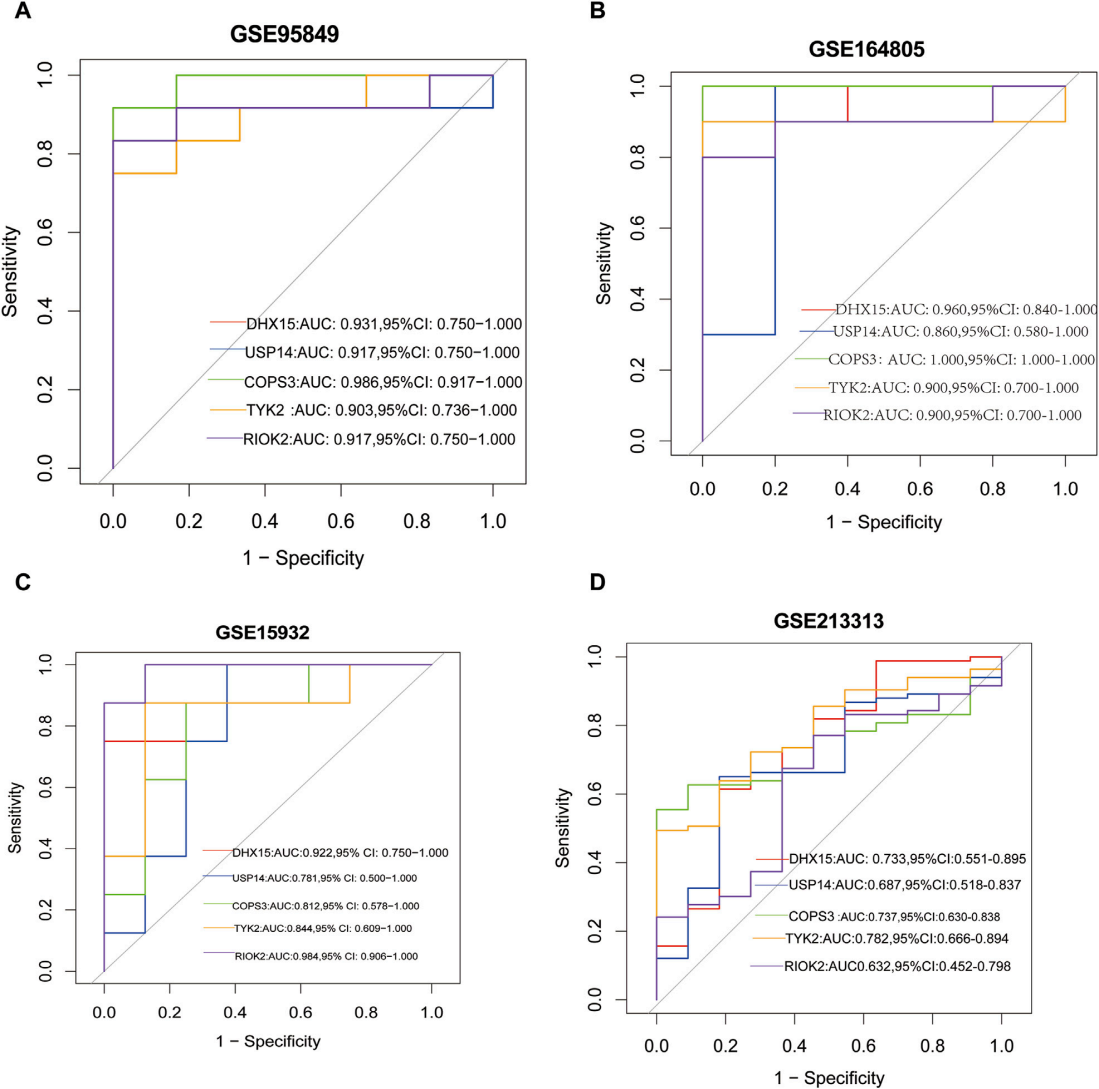
**(A)** The ROC curve of the diagnostic efficacy in GSE95849; **(B)** The ROC curve of the diagnostic efficacy in GSE164805; **(C)** The ROC curve of the diagnostic efficacy in GSE15932; **(D)** The ROC curve of the diagnostic efficacy in GSE213313.

We evaluated the diagnostic performance of the common feature genes for T2DM and COVID-19 by plotting ROC curves. The results from the ROC curve analysis showed that *DHX15*, *USP14*, *COPS3*, *TYK2*, and *RIOK* had AUC values of 0.931, 0.917, 0.986, 0.903, and 0.917, respectively, for T2DM (Figure 5A) and AUC values of 0.960, 0.860, 1.0, 0.9, and 0.90, respectively, for COVID-19 (Figure 5B). In the validation dataset, the AUC values of *DHX15*, *USP14*, *COPS3*, *TYK2*, and *RIOK* were 0.922, 0.781, 0.812, 0.844, and 0.984, respectively, for T2DM (Figure 5C), and 0.733, 0.687, 0.737, 0.782, and 0.632, respectively, for COVID-19 (Figure 5D). These results suggest that these five shared core genes can serve as effective diagnostic biomarkers for distinguishing between T2DM and non-diabetes and COVID-19 and healthy controls.

### 3.5 Interactome network of common feature genes and their co-expressed genes

**FIGURE 6:**
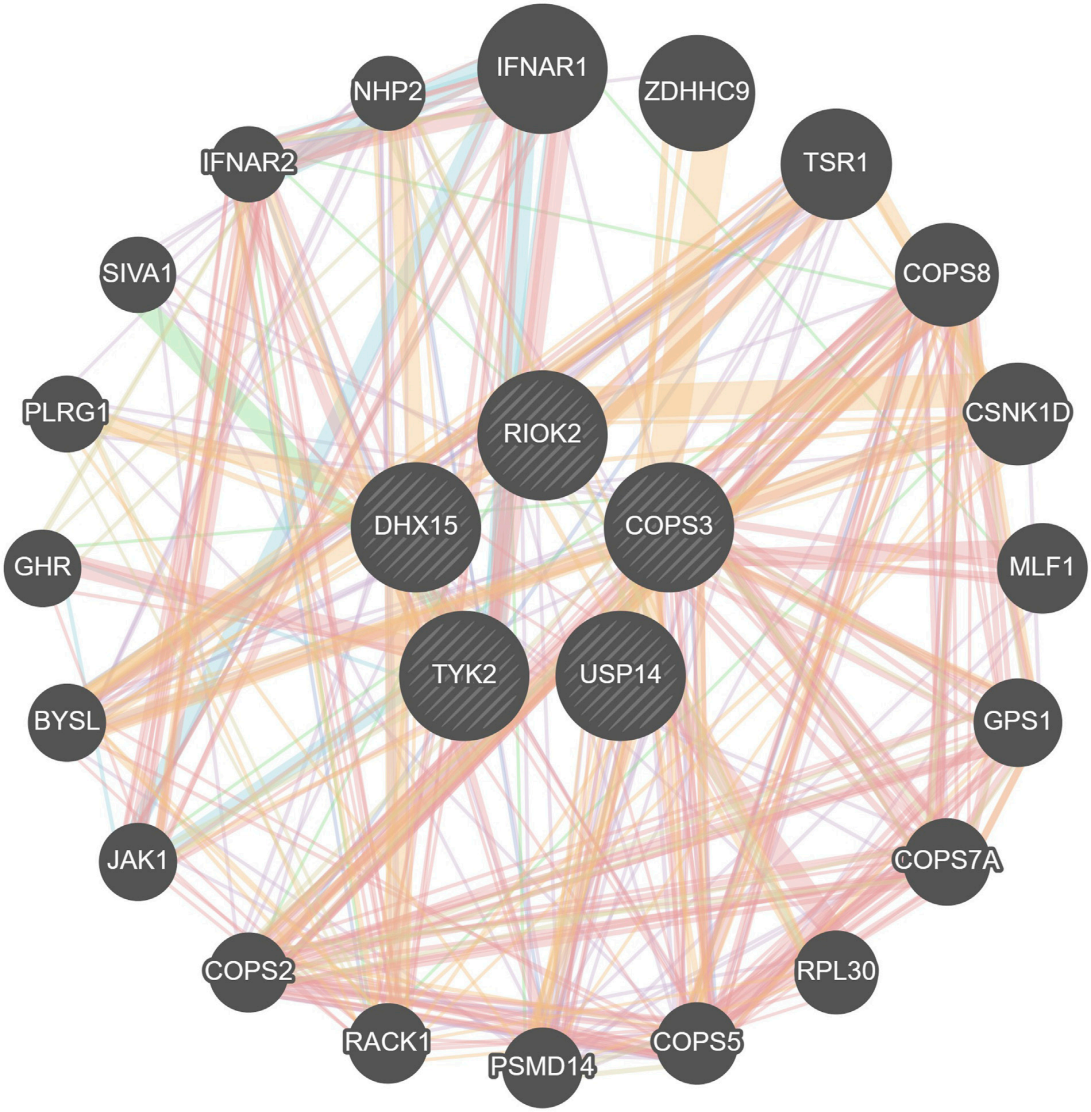
Interactome network of common feature genes and their co-expressed genes.

GeneMANIA analysis revealed a complex interactome network of common feature genes and their co-expressed genes between T2DM and COVID-19, with “physical interactions” accounting for 77.64%, “predicted” for 5.37%, “co-expression” for 8.01%, “co-localization” for 3.63%, “pathway” for 1.88%, “genetic interactions” for 2.87%, and “shared protein domains” for 0.60% of all interactions. The main biological functions of this network were related to processes such as protein modification by small protein removal, nucleotide-excision repair, and response to type I interferon (Figure 6; Supplementary Material). These results further emphasize the potential involvement of metabolism and inflammatory response and their associated pathways in the development and progression of both T2DM and COVID-19.

### 3.6 Correlation analysis of common feature genes

**FIGURE 7:**
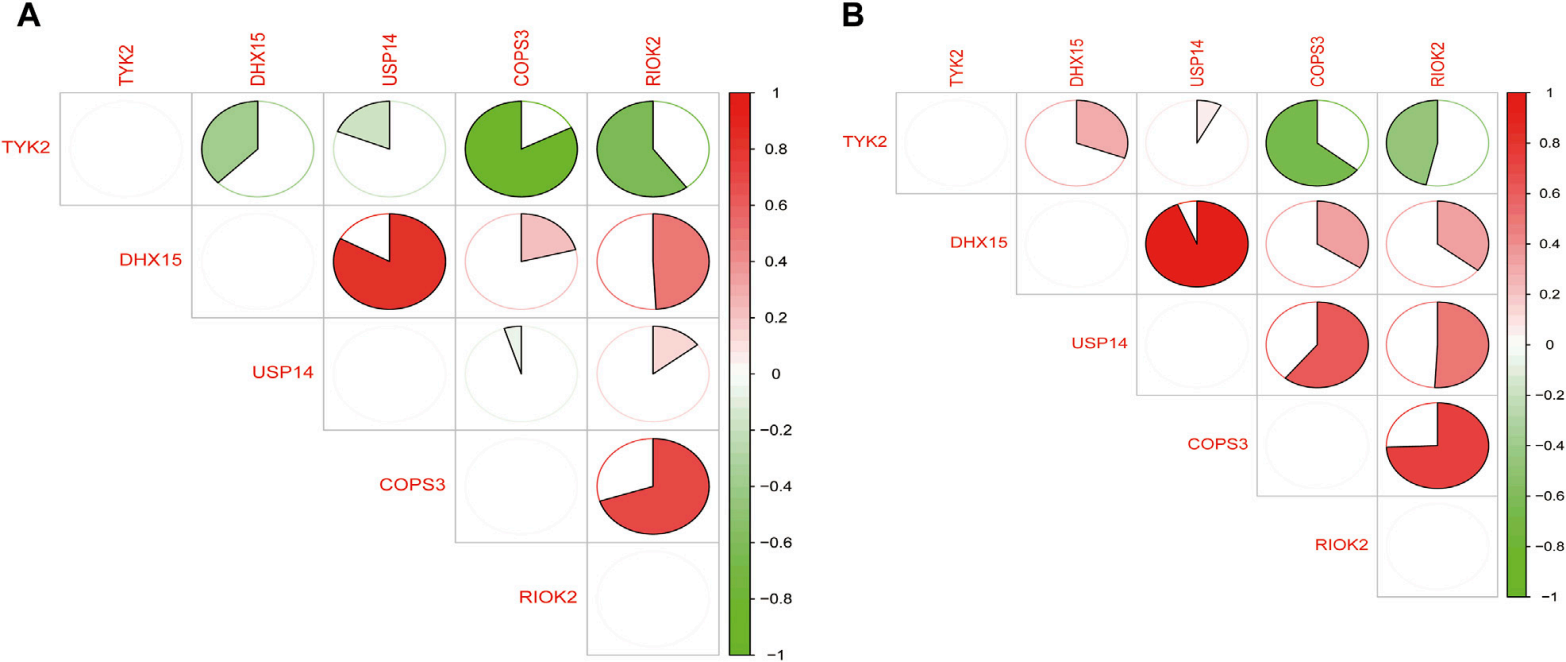
**(A)** Regulatory relationships of common feature genes in T2DM; **(B)** Regulatory relationships of common feature genes in COVID-19 (green represents negative correlation and red represents positive correlation).

We performed a correlation analysis of the common feature genes using the “corrplot” package in R. Through this analysis, we observed a significant positive correlation between *DHX15* and *USP14* expression, a significant positive correlation between *COPS3* and *RIOK2* expression, and a significant negative regulatory relationship between *TYK2* and *COPS3* as well as *RIOK2* expression. This regulatory trend was consistent in both T2DM and COVID-19 (Figure 7).

### 3.7 MiRNA-TF-mRNA regulatory network analysis

**FIGURE 8:**
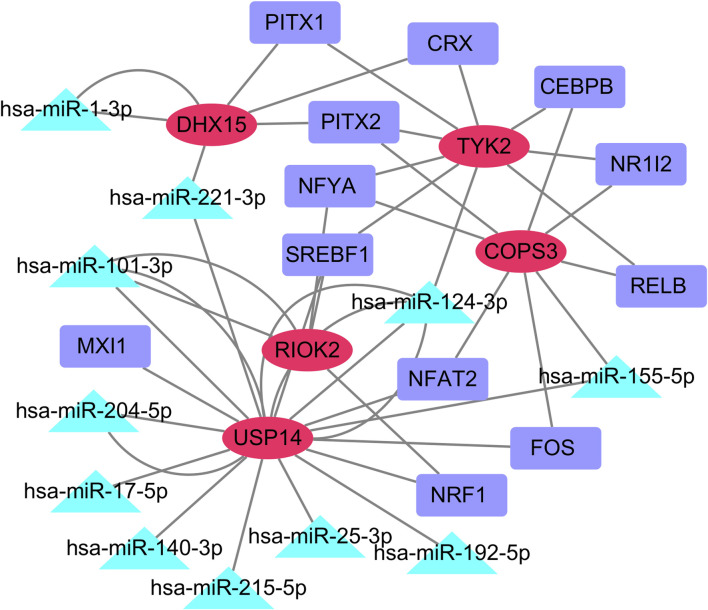
miRNA-TF-mRNA regulatory network. The red oval represents the common core genes, the green triangle represents the predicted miRNA, and the purple rectangle represents the predicted TFs. (Abbreviations: mRNA, messenger RNA; miRNA, microRNA; TF, transcription factor).

We retained all predicted miRNAs in miRTarBase, Starbase, and Targetsca, as well as all predicted TFs from the Enrich databases TRANSFAC and JASPAR PWMS for the common feature genes. Using Cytoscape, we visualized the miRNA-TF-mRNA regulatory network, which consisted of five common feature genes, 11 predicted miRNAs, and 12 predicted TFs (Figure 8).

We observed that TYK2 was targeted by PITX1, PITX2, CRX, NFYA, SREBF1, RELB, NR1L2, and CEBPB, whereas COPS3 was targeted by NR1L2, RELB, FOS, NFAT2, and NFYA. We also found that miR-124-3p simultaneously regulates THK2, RIOK2, and USP14; miR-221-3p regulates DHX15 and USP14; miR-101-3p regulates RIOK2 and USP14; and miR-155-5p regulates COPS3 and USP14.

### 3.8 Candidate drugs

We entered the common feature genes into the DGIdb website and obtained results for candidate drugs. The analysis revealed that the non-selective Tyk2 inhibitor, Baricitinib, had the highest overall score (query score of 2.15 and interaction score of 1.55), followed by the Janus kinase inhibitor Tofacitinib (query score of 1.84 and interaction score of 1.32) as potential drugs.

## 4 Discussion

Evidence suggests that patients with T2DM have a higher incidence, severity, and mortality rate of COVID-19. The mechanisms underlying this relationship are not fully understood but may be related to a dysregulated inflammatory response (Sardu et al., 2020; Nyland et al., 2021). There is limited research exploring the shared molecular mechanisms between T2DM and COVID-19 at the gene level. This study explored the molecular biological functions and pathways common to T2DM and COVID-19, providing clues for developing dual-purpose prevention and treatment strategies.

We analyzed transcriptome data of T2DM and COVID-19 blood samples and identified 221 shared DEGs. GO and KEGG analyses were performed on these DEGs. GO analysis showed that the shared DEGs were closely related to the negative regulation of phosphate and phosphorus metabolic processes, rRNA metabolic processes, positive regulation of mitotic nuclear division, T-cell co-stimulation, and lymphocyte co-stimulation. KEGG enrichment analysis indicated that these shared DEGs were predominantly involved in human T-cell leukemia virus 1 infection, type I diabetes, nucleotide sugar biosynthesis, Th1 and Th2 cell differentiation, and T-cell receptor signaling pathways. Many studies have shown that T-cell function is impaired in patients with T2DM. Our findings suggest that the shared molecular mechanisms between T2DM and COVID-19 may be related to dysregulated T-cell function and nucleotide metabolism. Further investigation of these pathways may provide a basis for developing new prevention and treatment strategies for both diseases (Moura et al., 2017). In obese patients with diabetes, there may be an elevation of activated CD4, CD278 co-stimulatory T-cells, Th17 cells, and cytotoxic T-cells (Richard et al., 2017). Th17 cells are critical in recruiting and invading microorganisms through immune cell recruitment and phagocytosis (Marqués et al., 2012). In addition, Farnsworth et al. reported that patients with T2DM exhibit defects in humoral immunity (Farnsworth et al., 2018). There is evidence to suggest that patients with T2DM exhibit decreased cytokine expression and impaired T-cell responses following viral stimulation (Richard et al., 2017; Tong et al., 2021). CD4 and CD8 T-cells have been detected in patients with COVID-19 (Grifoni et al., 2020; Tong et al., 2021). This suggests the importance of T-cell responses in COVID-19, and we speculate that the dysfunction of T-cells and lymphocytes in T2DM may affect the progression of COVID-19 disease.

We constructed a PPI network and identified five common feature genes (*DHX15*, *USP14*, *COPS3*, *TYK2*, and *RIOK*) of T2DM and COVID-19 by extracting the core genes of the network. We used ROC curve analysis to evaluate diagnostic performance, and the AUC values for T2DM diagnosis were 0.931, 0.917, 0.986, 0.903, and 0.917 for *DHX15*, *USP14*, *COPS3*, *TYK2*, and *RIOK2*, respectively. The AUC values for COVID-19 diagnosis were 0.960, 0.860, 1.0, 0.9, and 0.90, respectively. We also validated these results using independent T2DM and COVID-19 datasets without overlapping data with the training set, which showed good diagnostic performance. These findings suggest that *DHX15*, *USP14*, *COPS3*, *TYK2*, and *RIOK2* play important roles in developing T2DM and COVID-19.

DHX15 is a member of the RNA helicase DEAH-box family, which is widely expressed in immune cells (Mosallanejad et al., 2014; Detanico et al., 2019). DHX15 has both ATPase and helicase activities (Semlow et al., 2016; Bohnsack et al., 2022; Wang et al., 2022) and plays a role in various cellular processes, including pre-mRNA splicing and ribosomal RNA synthesis (Bohnsack et al., 2021). Most current research on DHX15 is focused on cancer and immunity. Mutations in the *SF3B1* splicing factor gene can disrupt the interaction between the splicing factor and SUGP1, leading to splicing errors that can cause cancer. DHX15, as an RNA helicase, is required for SUGP1 and participates in RNA error mediated by SF1B3 in cancer (Zhang et al., 2022). DHX15 forms aggregates with NLRP6 inflammasomes and dsRNA, which participates in host defenses (Shen C. et al., 2021). DHX15 regulates natural killer cell differentiation through the IL-15 signaling pathway, which depends on the ATPase activity of DHX15 rather than its RNA helicase activity. It is a crucial regulatory factor for the homeostasis and function of natural killer cells (Wang et al., 2022). Furthermore, DHX15 is essential in the context of mild/severe COVID-19 and its association with hepatocellular carcinoma and chronic hepatitis B (Sokouti, 2022). However, its ATPase activity and involvement in pre-mRNA splicing have been implicated in pancreatic beta-cell failure (Cnop et al., 2017; Herchuelz and Pachera, 2018). DHX15 may contribute to the progression of diabetes by affecting pancreatic beta cells.

Ubiquitin-specific protease 14 (USP14) is a deubiquitinating enzyme and a member of the ubiquitin-specific protease family. USP14 prevents protein substrate degradation by blocking the ubiquitin chain and promoting protein degradation by activating proteases (Wang F. et al., 2021). USP14 plays a role in the immune response by regulating signaling molecules involved in immunity (Wang F. et al., 2021; Wang D. et al., 2021). In addition to its role in the immune response, USP14 also plays an important role in viral infections, inflammation, tumors, autophagy, and neurodegenerative disorders (Wang D. et al., 2021; Li et al., 2021; Ming et al., 2022; Shi et al., 2022). USP14 is a potential therapeutic target for the treatment of α-herpesvirus-associated diseases. Lowering USP14 levels can inhibit viral replication, whereas agonizing USP14 restores viral replication (Ming et al., 2022). Inhibition of USP14 promotes autophagy in M1 macrophages, which reduces the severity of sepsis induced by cecal ligation and puncture (Xu et al., 2020). Thus, USP14 is closely related to metabolism, immunity, and inflammation. Research has shown that USP14 increases the levels and stability of 3′,5′-cyclic adenosine monophosphate response element-binding protein, enhancing the action of glucagon and hepatic glucose production, thereby promoting hyperglycemia (Liu et al., 2019). Additionally, the long non-coding RNA *OGRU* competes with miR-320 to regulate the expression of *USP14*, thereby mediating the progression of diabetic retinopathy (Fu et al., 2021). Furthermore, ubiquitination is crucial in virus infection by modifying viral proteins or host defense factors (Sun et al., 2019; Chen et al., 2024).

COP9 signalosome subunit 3 (COPS3) is an oncogene that promotes lung metastasis in osteosarcoma (Zhang et al., 2018). Autophagy induced by COPS3 promotes cisplatin resistance (Niu et al., 2023), and people with tumors with a low expression of COPS3 and Ras features have a better prognosis (Luo et al., 2009). COPS3 promotes the proliferation, invasion, and epithelial-to-mesenchymal transition of colorectal cancer cells through the MEK/ERK signaling pathway (Xie et al., 2022). It plays a crucial role in linking the Raf-1/MEK/ERK signaling pathway and autophagy regulation, inducing RAB7 to promote autophagosome and lysosome fusion (Zhang et al., 2018; Niu et al., 2023). The role of COPS3 in immunity is not yet clear. Research indicates that capsaicin and quercetin from chili peppers enhance hypoglycemic bioactivity through the RAS/Raf-1/MEK/ERK signaling pathway (Mi et al., 2023). Furthermore, studies suggest that autophagy plays a crucial role in maintaining the normal structure and function of pancreatic islets (Lee, 2014), and increased autophagy may delay the progression of T2DM and protect β-cell function (Yuan et al., 2017; Lambelet et al., 2018; Zhao et al., 2023a). Therefore, COPS3 may regulate the development of T2DM through the Raf-1/MEK/ERK signaling pathway and autophagy regulation. Additionally, SARS-CoV-2 can hinder autophagy by blocking autophagosome-lysosome fusion (Miao et al., 2021), and activation of autophagy can inhibit the replication and spread of SARS-CoV-2 (Gassen et al., 2021; Wei et al., 2021). Thus, COPS3 may regulate the development of COVID-19 by modulating autophagy.

Tyrosine kinase 2 (TYK2) is an intracellular kinase and a member of the Janus kinase family. TYK2 participates in the development of immune-related diseases by mediating cytokine signaling (Mease et al., 2022). The clinical efficacy of TYK2 inhibitors in inflammatory and autoimmune diseases has been demonstrated (Gonciarz et al., 2021), as evidenced by genomic, transcriptomic, and proteomic analyses detecting *TYK2* mutations and expression changes in various tumors (Hammarén et al., 2019). TYK2 plays a crucial role in the progression of both diabetes and COVID-19. TYK2 regulates apoptosis in pancreatic islet β-cells and innate immune responses (Marroqui et al., 2015; Chandra et al., 2022). Interestingly, reduced expression of the *TYK2* gene in pancreatic β-cells is a contributing factor to virus-induced susceptibility to diabetes (Izumi et al., 2015). The type I interferon response is one of three major pathways that affect susceptibility to and severity of COVID-19, and *TYK2* (located at 19p13.2) contains candidate pathogenic genes associated with the type I interferon pathway (Host Genetics Initiative, 2022). The *TYK2* locus shows a significant genetic association with severe COVID-19 (Pairo-Castineira et al., 2023). Defects in the *TYK2* gene lead to a weakened antiviral response in the body (Shimoda et al., 2000), and downregulation of *TYK2* is a molecular mechanism by which SARS-CoV-2 fails to induce a normal interferon response (Akbari et al., 2022).

RIOK2 belongs to the family of RIO kinases, which comprises three members: RIOK1, RIOK2, and RIOK3 (LaRonde-LeBlanc and Wlodawer, 2005). RIO kinases play a role in pre-ribosomal RNA processing and the biogenesis of ribosomes (Cerezo et al., 2021). The role of RIO kinases in cancer has been increasingly investigated, with the RIOK2 ATPase activity essential for cell survival. RIOK2 depletion leads to ribosome instability, decreased protein synthesis in leukemia cells, and apoptosis (Messling et al., 2022). The atypical kinase RIOK2 drives erythroid differentiation, inhibits megakaryocytic and myeloid formation, and plays an essential role in the transcriptional regulation of human hematopoietic differentiation. RIOK2 is closely associated with acute myeloid leukemia, myelodysplastic syndrome, and chronic kidney disease (Ghosh et al., 2022). RIO kinases regulate the cell cycle, AKT signaling, and activation of mutant RAS-driven tumor development (Asquith et al., 2019). Silencing RIOK2 inhibits the migration, invasion, and epithelial-mesenchymal transition of glioma cells, while overexpression of RIOK2 promotes these processes (Song et al., 2020). Currently, there is no research on the role of RIOK2 in T2DM. However, substance metabolism and biosynthesis are closely related to T2DM. Research reports suggest that RIOK is associated with host immune responses. RIOK3 inhibits the antiviral immune response by promoting degradation mediated by TRIM40 of RIG-I and MDA5 (Shen Y. et al., 2021; Zhao et al., 2023b). The immune response plays a crucial role in the progression of COVID-19 and T2DM. The roles of *DHX15*, *USP14*, *COPS3*, *TYK2*, and *RIOK2* in T2DM and COVID-19 remain primarily unknown, emphasizing the importance of future research.

Functional networks of shared genes and their co-expressed genes mainly focus on protein modification by removing small proteins, nucleotide excision repair, and response to type I interferon during viral infection. TFs in the host (including interferon regulatory factors and nuclear factor kappa B) are activated, recruiting specific subpopulations of white blood cells (Hur, 2019; Rajpal et al., 2020). Hyperglycemia, on the other hand, inhibits the production of IFN-1 (Lazear et al., 2019; Rajpal et al., 2020). The impact on the host’s defense against viral infections is likely to involve metabolic pathways and immune responses, which may contribute to the development and progression of both T2DM and COVID-19.

Using the miRTarBase, Starbase, and Targetscan databases, we successfully predicted the targeted miRNAs of the common core genes. We predicted TFs targeted using the Enrichr database to construct a miRNA-TF-mRNA regulatory network. Previous studies have shown that many of the predicted miRNAs are involved in the development of inflammation, immunity, diabetes, and COVID-19. For example, miR-124-3p inhibits high glucose-induced endothelial cell dysfunction (Zhao and He, 2021), whereas miR-1-3p induces endothelial cell dysfunction through targeting SERP1 (Gao et al., 2021). MiR-221 plays a role in diabetes and COVID-19. MiR-221-3p promotes diabetic wound healing by targeting HIPK285 and regulates microvascular dysfunction in diabetic retinopathy by targeting TIMP3 (Wang C. et al., 2020). Dexmedetomidine protects against renal fibrosis in diabetic mice through targeting the miR-101-3p-mediated endothelial-mesenchymal transition (Song et al., 2022). MiR-204-5p regulates endoplasmic reticulum stress in human pancreatic beta cells (Grieco et al., 2019) and reduces renal interstitial cell injury induced by high glucose by blocking the AKT/NF-κB pathway (Qiu et al., 2022). Cyanidin-3-O-glucoside protects endothelial cells by regulating the miR-204-5p/SIRT1-mediated inflammatory response and apoptosis (Wang Z. et al., 2020). MiR-17-5p inhibits the TXNIP/NLRP3 inflammatory pathway and promotes glucose uptake in HTR8/SVneo trophoblast cells (Jiang et al., 2022). Downregulation of miR-17-5p alleviates apoptosis and fibrosis in human renal interstitial cells induced by high glucose (Chen et al., 2021). High glucose also inhibits miR-140-3p, impairing vascular endothelial cell angiogenesis in diabetes (Wang et al., 2019). MiR-215-5p regulates Doxorubicin-induced myocardial cell injury through targeting ZEB2 (Xiong et al., 2021). MiR-25-3p activates autophagy and improves high glucose-induced podocyte injury by inhibiting DUSP1 expression (Huang et al., 2020). Decreased expression of miR-192-5p is associated with diabetic nephropathy (Akpınar et al., 2022). MiR-221 is involved in the inflammatory immune response of severe COVID-19 patients (Gaytán-Pacheco et al., 2022). MiR-101-3p and miR-25-3p exhibit differential expression between severe/critical COVID-19 patients and patients with mild infections (Nicoletti et al., 2022). MiR-140-3p shows differential expression in lung tissues before and after COVID-19 infection (Kim et al., 2020). Significant differences in miR-192-5p expression exist between surviving and deceased patients with severe COVID-19 (de Gonzalo-Calvo et al., 2021). Additionally, miR-320 regulates USP14 expression and mediates the progression of diabetic retinopathy (Fu et al., 2021). MiR-26a-5p regulates the USP14/NF-κB pathway to alleviate inflammation and oxidative stress in diabetic retinopathy (Bian et al., 2024).

In summary, identifying common core genes and pathways in T2DM and COVID-19 may provide new insights into potential therapeutic targets for patients with both conditions. However, common biomarkers, pathways, and therapeutic drugs need to be confirmed through experiments. In this study, we identified common DEGs and common feature genes in T2DM and COVID-19 through bioinformatics analysis methods, predicted miRNAs, and TFs targeting commonly expressed genes.

Our findings suggest that T2DM and COVID-19 may share common pathogenic mechanisms mediated by specific hub genes. This study provides novel avenues for further molecular mechanism research.

Limitations of this study include the need for further experimental validation of the common core genes and pathways identified in T2DM and COVID-19. In addition, the diagnosis of T2DM and COVID-19 cannot be based solely on these common feature genes and pathways but requires consideration of symptoms and laboratory tests.

## 5 Conclusion

We identified five common feature genes (*DHX15*, *USP14*, *COPS3*, *TYK2*, and *RIOK2*) and their co-regulatory pathways between T2DM and COVID-19, which may provide new insights for molecular mechanism studies.

## Data Availability

Publicly available datasets were analyzed in this study. This data can be found here: https://www.ncbi.nlm.nih.gov/geo/.

## Abbreviations

T2DM, type 2 diabetes mellitus; COVID-19, coronavirus disease 2019; DEG, differentially expressed genes; GO, gene ontology; KEGG, Kyoto Encyclopedia of Genes and Genomes; PPI, protein-protein interaction; ROC, receiver operating characteristic; AUC, area under the curve; BP, biological process; MF, molecular function; CC, cellular component.
