# Supplementary figures and images for: Identification of common genes and pathways between type 2 diabetes and COVID-19
Source: Front Genet. 2024 Apr 18;15:1249501. doi: 10.3389/fgene.2024.1249501 (PMC11063347; doi:10.3389/fgene.2024.1249501)

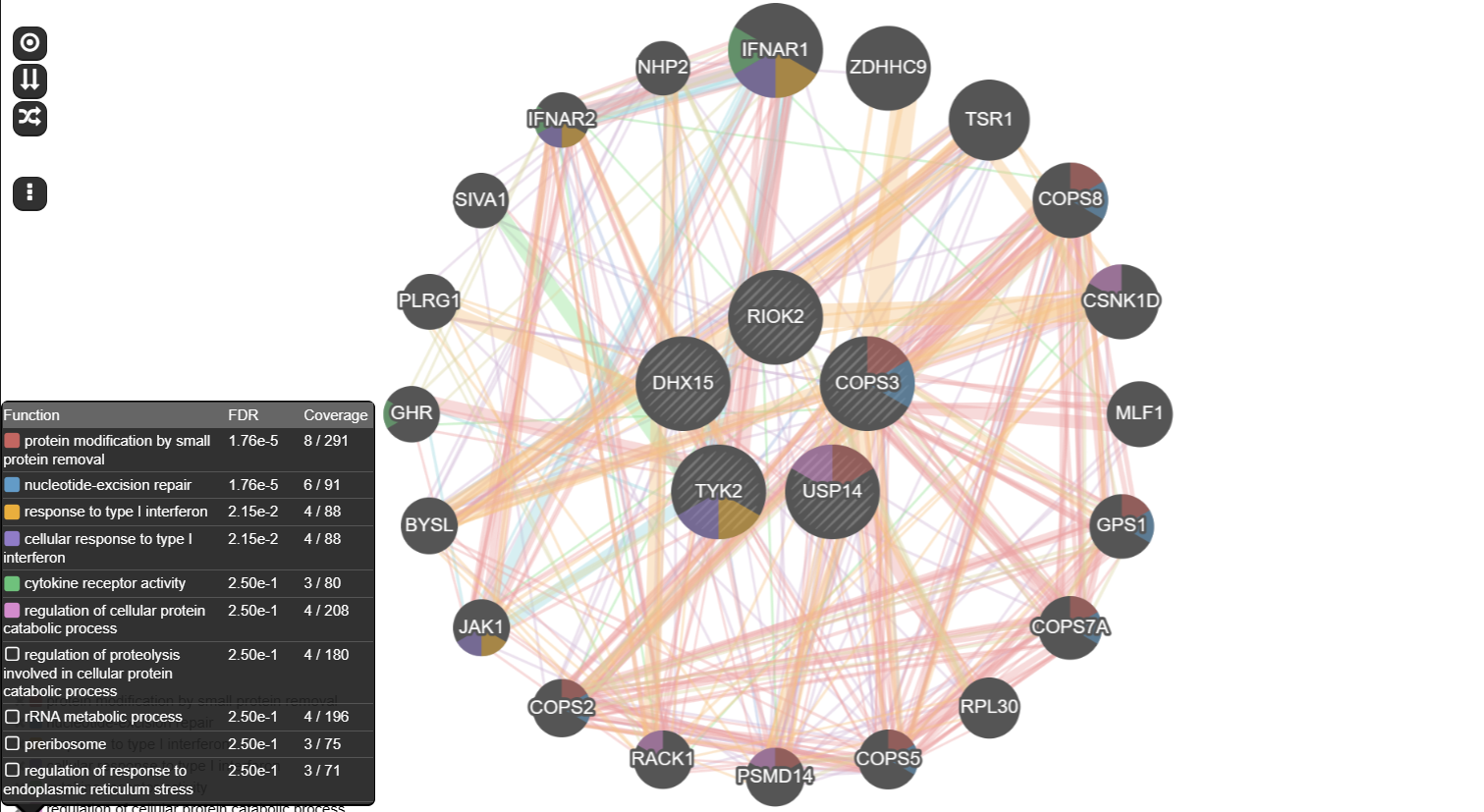

Supplement: Supplementary file 1 [file DataSheet1.ZIP › 补充材料/genemania.png]
